# Symptom Clusters in People Living with HIV Attending Five Palliative Care Facilities in Two Sub-Saharan African Countries: A Hierarchical Cluster Analysis

**DOI:** 10.1371/journal.pone.0126554

**Published:** 2015-05-12

**Authors:** Katrien Moens, Richard J. Siegert, Steve Taylor, Eve Namisango, Richard Harding, ENCOMPASS, EURO IMPACT

**Affiliations:** 1 King's College London, Cicely Saunders Institute, Department of Palliative Care, Policy & Rehabilitation, London, United Kingdom; 2 Auckland University of Technology, Auckland, New Zealand; 3 African Palliative Care Association, Kampala, Uganda; Hospital Italiano de Buenos Aires, ARGENTINA

## Abstract

**Background:**

Symptom research across conditions has historically focused on single symptoms, and the burden of multiple symptoms and their interactions has been relatively neglected especially in people living with HIV. Symptom cluster studies are required to set priorities in treatment planning, and to lessen the total symptom burden. This study aimed to identify and compare symptom clusters among people living with HIV attending five palliative care facilities in two sub-Saharan African countries.

**Methods:**

Data from cross-sectional self-report of seven-day symptom prevalence on the 32-item Memorial Symptom Assessment Scale-Short Form were used. A hierarchical cluster analysis was conducted using Ward’s method applying squared Euclidean Distance as the similarity measure to determine the clusters. Contingency tables, X^2^ tests and ANOVA were used to compare the clusters by patient specific characteristics and distress scores.

**Results:**

Among the sample (N=217) the mean age was 36.5 (SD 9.0), 73.2% were female, and 49.1% were on antiretroviral therapy (ART). The cluster analysis produced five symptom clusters identified as: 1) dermatological; 2) generalised anxiety and elimination; 3) social and image; 4) persistently present; and 5) a gastrointestinal-related symptom cluster. The patients in the first three symptom clusters reported the highest physical and psychological distress scores. Patient characteristics varied significantly across the five clusters by functional status (worst functional physical status in cluster one, p<0.001); being on ART (highest proportions for clusters two and three, p=0.012); global distress (F=26.8, p<0.001), physical distress (F=36.3, p<0.001) and psychological distress subscale (F=21.8, p<0.001) (all subscales worst for cluster one, best for cluster four).

**Conclusions:**

The greatest burden is associated with cluster one, and should be prioritised in clinical management. Further symptom cluster research in people living with HIV with longitudinally collected symptom data to test cluster stability and identify common symptom trajectories is recommended.

## Background

Symptoms are more often clinically presented in advanced stages of HIV [1]. Fatigue, anorexia, pain, dyspnoea, insomnia, diarrhoea, depression, anxiety and worry each have a prevalence of at least 50% in advanced HIV [2]. Efficient symptom control is achieved by detailed and regular symptom assessment.

Clinical research has historically focussed on single symptoms [3]. This over-emphasis on the study of single symptoms has limited the understanding of the burden of multiple symptoms [3]. Recently, clinical research has begun to study the interaction among symptoms [4], which has led to the use of the term “symptom cluster” to describe a group of three or more related symptoms [5]. The current evidence provides a large number of studies on symptom clustering in people living with cancer [6]. To date one study has identified symptom clusters in people living with HIV, and this study did not have the derivation of symptom clusters as its primary outcome but used data prior to antiretroviral therapy (ART) availability and sought to establish underlying factors in symptom measurement [7].

Symptom cluster studies are important to inform the management of symptoms to help lessen the total symptom burden through appropriate treatment plans [6]. For example, symptoms belonging to a specific cluster may have a common biological mechanism for which the treatment of one symptom can result in the alleviation of the other symptoms of that cluster [8]. Knowledge of symptom clusters can prompt clinical investigation of associated symptoms when one symptom is detected. There is evidence that this is true for gastrointestinal [9] and psychological [10] symptoms.

There are two different approaches to conducting a symptom cluster analysis: a clinical and a statistical one [11]. A clinical symptom cluster analysis approach is used in studies that focus on a small number of preselected symptoms and they mostly use correlation methods [8]. The starting point for this type of symptom cluster analysis is clinically observed associations of symptoms that are likely to cluster such as pain, fatigue, and depression [8]. A statistical approach is generally applied in studies using multi-symptom assessment tools or symptom checklists that include a large number of symptoms [8]. The starting point for this type of analysis is that there is no clinical assumption of symptom associations. Factor or cluster analyses are used to discover statistically defined symptom clusters. The statistical analysis of multiple symptoms can identify clusters that may have been overlooked in clinical assessment [6]. For this reason, statistically derived symptom clusters appear to be more objective than clinical clusters [6], and may be useful to inform clinical guidelines.

The study and management of symptoms among people living with HIV is essential, as evidence shows that symptom burden is associated with a number of key outcomes [12]. These are firstly sexual risk taking [13], second poor adherence to ART [14], third treatment switching [15], fourth viral rebound [16], fifth poorer quality of life [17], and sixth suicidal ideation [18].

The aim of this study was to employ a statistical cluster analysis technique to identify and compare symptom clusters among people living with HIV attending five palliative care facilities in two sub-Saharan African countries. The objectives were: 1) to identify clusters of subjects with similar combinations of symptoms; 2) to describe the symptom combinations that distinguish the clusters; and 3) to compare the clusters with reference to treatment, demographic and symptom distress related characteristics.

## Methods

### Study design

This study was part of an international, multicenter, cross-sectional study using the Memorial Symptom Assessment Scale-Short Form (MSAS-SF) to establish the seven-day prevalence and associated burden of physical and psychological symptoms of HIV patients receiving palliative care in sub-Saharan Africa [19].

### Setting and sample

The study took place in four palliative care facilities in South Africa and one in Uganda. Palliative care was provided as home care, day care, hospital consulting, and palliative inpatient services, and provide holistic family-based care from diagnosis to the end of life and into bereavement. Referrals are taken from treatment centres so that palliative care can be provided alongside ART.

The inclusion criteria for the respondents were: being an adult with a documented HIV positive sero status according to the health facility records and known to the patient; under palliative care, and satisfactory physical and cognitive ability to participate in the interviews.

For this analysis HIV patients with a cancer diagnosis were excluded.

### Measurement

The MSAS-SF is a 32-tem inventory that assesses the symptoms over a seven-day period prevalence. This multidimensional instrument aims to capture the presence and distress of 26 physical and six psychological symptoms. The associated distress of the 32 symptoms can be scored on three indices: Physical Symptom Distress Index (MSAS-Phys), Psychological Symptom Distress Index (MSAS-Psych), and Global Distress Index (MSAS-GDI). These three distress-subscales are scored on a range of 0 (least distressed)- 4 (most distressed). The MSAS-SF possesses good psychometric properties e.g. subscale Cronbach’s alpha coefficients of 0.76–0.87 indicate good internal consistency, and there is the proven one-day test-retest reliability correlation coefficients of 0.86–0.94 [20]. The MSAS-SF has been regularly applied among African HIV populations [21–26].

Research nurses read aloud the MSAS-SF questions and recorded patient responses due to the potential limited literacy among the respondents, and to reduce bias of mixed completion methods. As reported in the paper on prevalence from this study [19], all questionnaires were translated and implemented in local languages (Luganda, Runyankole, SeSotho, Runyoro, SeTswana, isiXhosa and isiZulu Gauteng and KwaZulu Natal dialects).

### Analysis

To describe and conduct our cluster analysis the data of the following variables were extracted: gender (male/ female); age (continuous variable); being on antiretroviral ART (yes or no); physical functional status using the grading of the Eastern Cooperative Oncology Group (ECOG) scoring system [27] (fully active—restricted—ambulatory—limited self-care—completed disabled); presence of 32 MSAS-SF symptoms (present/ not present), and the PSYCH, PHYS and GDI distress scores (least distressed—most distressed).

First we described the sample by country on demographic and symptom data. As it has been reported in other studies in the region of people living with HIV disease [28], there were too many missing data for the CD4 count variable, therefore the ECOG was used as a measure of disease severity. The percentages of the patients with ‘limited self-care’ and ‘completely disabled’ scores were aggregated to represent the most severely physically impaired patients, as symptoms and ensuing functional impairment are most directly associated with the biological processes of a disease [29].

To develop an optimum solution, a two-stage sequence of analysis was followed. First, a hierarchical cluster analysis using Ward’s method applying squared Euclidean Distance as the distance or similarity measure was executed [30]. This helped to determine the optimum number of clusters to work with. Second, the hierarchical cluster analysis was rerun with the selected number of clusters, which enabled every case to be allocated to a particular cluster.

A hierarchical cluster analysis is a statistical method for finding comparatively homogenous clusters of cases based on measured characteristics (in this study: the presence of symptoms). This type of analysis starts with each case as a separate cluster i.e. there are as many clusters as cases, and then combines the clusters sequentially, reducing the number of clusters at each step. The clustering method uses the dissimilarities or distances between objects when constructing the clusters. To measure the distance between objects, the squared Euclidean distance measure was selected because it is a measure prescribed to use for dichotomous variables (in this study symptoms were scored dichotomously recorded only as ‘present’ or ‘not present’). In comparison to the Euclidean distance measure the squared Euclidean distance measure is used to place progressively greater weight on objects that are further apart. Ward’s method was used as the clustering algorithm i.e. the rules that regulate between which point distances are measured to determine cluster membership. The statistical software SPSS v.19.0 provides five clustering algorithms but Ward’s method was selected because it is a very efficient method, using an analysis of variance to evaluate the distance between clusters. Cluster membership is appraised by calculating the total sum of squared deviations from the mean of a cluster. The criterion for fusion is that it should produce the smallest possible increment in the error of the sum of squares [30]. For this study a hierarchical cluster analysis was conducted using three scenarios of combinations of four, five and six clusters. The dendrogram of each hierarchical cluster analysis was evaluated to determine which symptom needed to be allocated to which cluster.

To compare the clusters by patient specific characteristics (sex, being on ART and functional status) contingency tables and X^2^ tests (significance level: p<0.05) were used. To test for differences across the clusters on the level of age, GDI, PHY and PSYCH distress scores ANOVA (significance level: p<0.05) was used. The prevalence of the symptoms in each constructed cluster was also calculated.

The literature indicates that data on 224 patients are sufficient to conduct a hierarchical cluster analysis[11] i.e. for the study this meant that the data from the patients from the four palliative care facilities in South-Africa (177 patients) were merged together with the data from the patients in the palliative care facility in Uganda (47 patients). Also, including data from two separate locations improves the inference about the wider population (i.e. people of Africa living with HIV/ AIDS who are in palliative care), better than a single location even with the same sample size. Contingency tables and X^2^ tests (significance level: p<0.05) and ANOVA (significance level: p<0.05) were used to identify significant differences between countries.

The statistical software IBM SPSS Statistics v.19.0 was used for all statistical computations.

### Ethics

The study was approved by the Ethical Review Boards of the Universities of Cape Town, KwaZulu Natal, and Witwatersrand; the Ugandan National Council for Science and Technology; Hospice Africa Uganda; and the Hospice Palliative Care Association of South Africa. The consent forms and all other information approved by the Ethical Review Boards were translated from English into the principle languages of the regions where the respondents live. Signed informed consent was obtained from each respondent.

## Results

### Sample characteristics

The patients in the samples from South-Africa and Uganda are described separately in Table 1. This table shows that there was no difference in psychological symptom burden, but that physical symptom burden was slightly higher in South Africa and that this difference is then reflected in the global symptom burden.

**Table 1:** Characteristics of HIV+ patients in 4 South-African palliative care facilities (N = 177) and in one palliative care facility in Uganda (N = 47).

Characteristics		South-Africa		Uganda	
Place of receiving palliative care	At home	70*		53*	
	Outpatient	**-**		38*	
	Inpatient	25*		-	
	Day care	5*		9*	
Age (mean; S.D.)		36.3; 8.8		36.8; 10.0	
Sex: female (%)		77^†^		60^†^	
On ART (%)		48		53	
Functional status: limited self-care/ completely disabled (%)		32^†^		23^†^	
MSAS-SF symptoms and prevalence of symptoms:		Difficulty concentrating: 33*	Mouth sores: 17	Difficulty concentrating: 64*	Mouth sores: 23
		Pain: 80^†^	Problems with sexual interest/ activity: 31*	Pain: 94^†^	Problems with sexual interest/ activity: 11*
		Lack of energy: 71	Itching: 56	Lack of energy: 75	Itching: 70
		Cough: 60	Lack of appetite: 41	Cough: 49	Lack of appetite: 41^‡^
		Changes in skin: 51^‡^	Dizziness: 47^†^	Changes in skin: 64	Dizziness: 64^†^
		Dry mouth: 64^‡^	Difficulty swallowing: 18	Dry mouth: 51	Difficulty swallowing: 23
		Nausea: 39	Changes in way food tastes: 28	Nausea: 38	Changes in way food tastes: 36^§^
		Feeling drowsy/ tired: 75	Weight loss: 60	Feeling drowsy/ tired: 72	Weight loss: 62
		Numbness/ tingling in hands or feet: 66	Hair loss: 25^‡^	Numbness/ tingling in hands or feet: 72	Hair loss: 26
		Difficulty sleeping: 50	Constipation: 32	Difficulty sleeping: 47	Constipation: 34
		Feeling bloated: 35	Swelling of arms or legs: 27	Feeling bloated: 38	Swelling of arms or legs: 39^‡^
		Problems urinating: 34	“I don’t look myself”: 63*	Problems urinating: 30	“I don’t look myself”: 40*
		Vomiting: 20	Feeling sad: 76	Vomiting: 28	Feeling sad: 72
		Shortness of breath: 40	Worrying: 75^‡^	Shortness of breath: 26	Worrying: 66
		Diarrhoea: 24	Feeling irritable: 74^†^	Diarrhoea: 26	Feeling irritable: 54^‡^ ^†^
		Sweats: 55	Feeling nervous: 51	Sweats:60	Feeling nervous: 36
Level of symptom distress: (mean; S.D)	PSYCH	1.6; 0.9^‡^		1.3; 0.9^‡^	
	PHYS	1.5; 0.8^§^ *		1.3; 0.7^§^ *	
	GDI	1.8; 0.8^ǁ^ *		1.4; 0.7^§^ *	

Abbreviations: MSAS-SF = Memorial Symptom Assessment Scale-Short Form ART = Antiretroviral Therapy PSYCH = Psychological Symptom Subscale score PHYS = Physical Symptom Subscale score GDI = Global Distress Index score S.D. = Standard Deviation

*Significant at 0.001 level

^†^Significant at 0.05 level

^‡^Missing value = 1

^§^Missing value = 2

^ǁ^ Missing value = 3

A cluster analysis was eventually run on 217 cases because there were seven cases missing from the total sample of 224 cases. Each case responded to items on the presence of 32 symptoms that were assessed using the MSAS-SF. This sample of people had a mean age of 36.5 (SD = 9.0) of which 73.2% were female, and 49.1% currently on ART.

### Symptom clusters

The hierarchical cluster analysis produced five symptom clusters that fitted (Table 2). Cluster one included 40 patients, with 45% of them being severely physically impaired (i.e. patients who have limited self-care to being completely disabled as assessed by ECOG functional status scale), and it is characterised by symptoms reflecting severe skin problems (i.e. the ‘dermatological-related’ cluster). The second symptom cluster included 46 patients, with 37% of them being severely physically impaired, and it is characterised by symptoms that indicate the presence of a general feeling of anxiety together with problems with urinal and faecal elimination (i.e. the ‘generalised anxiety and elimination’ cluster). The third symptom cluster included 31 patients, with 32% of them being severely physically impaired, and it is characterised by symptoms influencing social interactions (including hair loss, mouth sores, swelling of arms or legs, diarrhoea, problems with sexual interest/ activity, difficulty swallowing, named the ‘social and image-related’ cluster). The fourth symptom cluster included 70 patients, with 23% of them being severely physically impaired, and it is characterised by symptoms that can be considered as persistently present throughout the disease trajectory of people living with HIV e.g. numbness, pain and cough (named the ‘persistently present’ cluster). The fifth symptom cluster included 30 patients, with 17% of them being severely physically impaired and it is characterised by symptoms indicating problems with the gastrointestinal system e.g. nausea and vomiting (named the ‘gastrointestinal-related’ cluster). In Table 2 all the psychological-related symptoms are underlined to distinguish them from physical symptoms and highlight their distribution across the symptom clusters.

**Table 2:** Characteristics of 5 symptom clusters using hierarchial cluster analysis among HIV^+^ palliative care patients (N = 217).

Characteristics of each cluster:		CLUSTER 1 ‘Dermatological- related’		CLUSTER 2 ‘Generalised anxiety & elimination‘		CLUSTER 3 ‘Social and image-related’		CLUSTER 4 ‘Persistently present‘		CLUSTER 5 ‘Gastro intestinal- related‘	
Ranking variable: functional status: limited self-care/ completely disabled (%)		45*		37*		32*		23*		17*	
Characteristics of patients in the cluster:	number of patients (n)	40		46		31		70		30	
	age (mean; S.D.)	36.4; 8.6		37.4; 10.1		36.3; 7.5		36.2; 8.8		36.0; 10.3	
	sex: female (%)	68.0		71.7		81.0		69.0		83.3	
	on ART (%)	45.0^†^		63.0^†^		65.0^†^		47.1^†^		27.0^†^	
MSAS-SF symptoms and prevalence of symptoms in the cluster:		1)itching	70.0	1)feeling nervous	76.1	1)swelling of arms or legs	29.0	1)numbness/ tingling in hands or feet	62.9	1)nausea	13.3
								2)pain	61.4		
				2)dizziness	41.3			3)feeling irritable	57.1		
		2)changes in skin	65.0	3)shortness of breath	37.0	2)hair loss	25.8	4)cough	52.9	2)vomiting	13.3
				4)feeling bloated	37.0			5)feeling drowsy/ tired	50.0		
				5)difficulty concentrating	36.9	3)mouth sores	22.6	6)worrying	45.7		
				6)difficulty sleeping	32.6			7)dry mouth	44.3	3)lack of appetite	6.7
								8)sweats	41.4		
				7)problems urinating	17.4	4)problems with sexual interest/ activity	22.6	9)feeling sad	40.0		
								10)lack of energy	38.6	4)changes in way food tastes	3.3
				8)constipation	17.4	5)diarrhoea	12.9	11)weight loss	28.6		
						6)difficulty swallowing	6.5	12)”I don’t look like myself”	28.6		
Level of distress in MSAS—SF symptom cluster:	PSYCH (mean; S.D.)	2.0; 0.8*		1.8; 0.7*		2.0; 0.7*		0.9; 0.8*		1.6; 0.7*	
	PHYS (mean; S.D.)	2.3; 0.7*		1.7; 0.6* ^‡^		1.5; 0.7*		0.8; 0.7*		1.2; 0.5*	
	GDI (mean; S.D.)	2.3; 0.7*		2.1; 0.6* ^‡^		2.1; 0.7*		1.1; 0.7*		1.5; 1.0*	

Abbreviations: MSAS-SF = Memorial Symptom Assessment Scale-Short Form ART = Antiretroviral Therapy PSYCH = Psychological Symptom Subscale score PHYS = Physical Symptom Subscale score GDI = Global Distress Index score S.D. = Standard Deviation

*Significant at 0.001 level

^†^Significant at 0.05 level

^‡^Missing value = 1

Table 2 also demonstrates that patient characteristics varied significantly across the five clusters by functional status; ART status; global distress subscale, physical distress subscale and psychological distress subscale. The highest proportion of physically impaired patients as measured by the ECOG scale were present in symptom cluster one the ‘dermatological related’ (45%), and the lowest proportion were present in cluster five the ‘gastrointestinal related’ symptom cluster (17%) (p<0.001). The highest proportion of patients who were on ART were present in symptom clusters two ‘generalised anxiety and elimination (63%)’ and three ‘social and image-related’ (65%), and the lowest proportion in cluster five the ‘gastrointestinal-related’ symptom cluster (27%) (p = 0.012).

Psychological burden was greatest in clusters one and three (‘dermatological-related’ and the ‘social aware and image-related’), and lowest in symptom cluster four the ‘persistent present’ symptom cluster (F = 21.8, p<0.001). The physical burden was greatest in symptom cluster one the ‘dermatological related’, and lowest in symptom cluster four the ‘persistent present’ symptom cluster (F = 36.3, p<0.001). The global distress burden was greatest in symptom cluster one the ‘dermatological-related’, and lowest in symptom cluster four the ‘persistent present’ symptom cluster (F = 26.8, p<0.001).

## Discussion

The results of this study show that among people living with HIV and receiving palliative care in five palliative care facilities in two sub-Saharan African countries five distinct statistically derived symptom clusters can be distinguished, with a clear differentiation between clusters.

The data on functional status, ART status, prevalence of the symptoms and the level of physical and psychological distress in each symptom cluster facilitate the determination of the relative clinical importance of each. The first three symptom clusters showed the highest level of distress among the respondents. Firstly, the ‘dermatological-related’ symptom cluster had the highest level of physical distress and also the highest proportion of physically impaired people. This may indicate that the patients belonging to this cluster were in a more advanced stage of the disease. The frequency of skin disorders increases with progressive immunodeficiency and may be more severe and respond less well to therapy [1]. The itching and the changes in skin can be caused by skin infections; skin disorders; hypersensitivity reactions; arthropathy, HIV polyarthritis, HIV arthropathy, septic arthritis, Reiter’s syndrome, or psoriatric arthritis.

Second, the ‘generalized anxiety and elimination-related’ symptom cluster had the second highest level of psychological distress and the highest proportion of people currently on ART. Cluster two could indicate the interaction between physical and psychological related problems, in that inability to control elimination may cause great distress. This cluster contained two psychologically related problems with ‘feeling nervous’ presenting with a prevalence of 76.1%. The other six symptoms that were present in the cluster are elimination related problems e.g. constipation. This cluster could illustrate the debilitating effect of living with an HIV diagnosis and the associated social, psychological and physical problems. Anxiety is highly prevalent among people living with HIV [31,32], and should be addressed swiftly, as the evidence states that unaddressed psychological needs have potentially severe clinical implications such as poor adherence [33].

Third, the ‘social and image-related’ symptom cluster was (together with cluster one) associated with the highest level of psychological distress. The highest proportion of patients currently on ART was also in this cluster. This cluster is composed of symptoms that influence the image of a person e.g. hair loss, swelling of arms and legs, and interpersonal problems such as decreased sexual interest/ activity; mouth sores and difficulty swallowing that would affect eating. The social dimension of these problems therefore can be easily understood to cause greater psychological burden, and as ART rollout is increased and HIV is seen as a chronic disease, the social dimensions of health and wellbeing (and the associated individual burden) must be understood in the context of continuing stigmatization.

We note several limitations in the interpretation of this novel data. Firstly, while this provides important new understanding on symptom clustering among HIV/ AIDS patients receiving palliative care (where the assessment and management of symptoms are most important) it may not be readily generalizable to all those with HIV disease. Second, as ART is rolled out the exploration of burden among those on ART may require specific study, as problems have been shown to persist after treatment initiation [31]. However, given prior global evidence that ART is not associated with self-report symptom burden [26,34], it was appropriate to include those both currently taking ART and those who are not, and to determine proportions of these groups in each cluster. Thirdly, given the unpredictable nature of HIV disease, it is unclear how stable this symptom clustering is over time, particularly as burden may become greater with long time on treatment [35], Fourth, given that we needed to apply the standard exclusion criterion of cognitive impairment (to avoid any potential bias through mixed self and proxy report), and we excluded those with cancer to make a more readily interpretable cluster structure, further study is required. Fifth, as CD4 counts were often missing (which is a problem reported in a similar study in outpatient settings) [28]) data on the physical functional status of the respondents were used as a proxy indicator of the disease progression. Sixth, the cross-sectional design meant that an evolution of the symptom clusters over time, and whether clusters are stable could not be determined. Seventh, a replication of the five clusters independently was not executed. Eighth, a cluster analytical approach does not allow us to understand the etiology of the clusters.

## Conclusion

This is the first study of symptom clusters in HIV/ AIDS patients receiving palliative care using a cluster analytical approach. Symptom clusters in this patient group could be identified and these are interpretable. These results also indicate that HIV/ AIDS patients who are on ART still show a high prevalence of symptoms causing a high level of distress. Given the high prevalence of psychosocial problems and stigma reported alongside ART [31], these data also offer important understanding of the co-existence of physical and psychological problems within symptom clusters, and therefore the patient must be assessed holistically.

These clusters demonstrate that assessment and treatment planning for symptom clusters should take account of their physical, psychological and social burden. From clinical and public health perspectives, assessment and control for people living with HIV are important because symptom burden is associated with sexual risk taking [13], poor adherence to ART [14], treatment switching [15], viral rebound [16], poorer quality of life [17], and suicidal ideation [18].

These findings are useful for directing assessment and care in clinical practice. The data demonstrate that patients in symptom cluster one (i.e. the ‘dermatological-related’ symptom cluster) require the greatest attention to their symptom burden, and that as function declines their burden is likely to increase.

Future research should investigate symptom clusters in people living with HIV in different stages of the disease trajectory, and longitudinal study designs may detect changes over time in symptom cluster constructs.

Importantly, the distress associated with symptom burden should be managed in routine HIV care.
